# Persistent and Progressive Exfoliative Dermatitis: A Case Report

**DOI:** 10.5811/cpcem.53897

**Published:** 2026-07-28

**Authors:** Jennifer Boukouris, Sherif Elsherif

**Affiliations:** Henry Ford Health Providence Hospital, Department of Emergency Medicine, Novi, Michigan

**Keywords:** exfoliative dermatitis, erythroderma, crusted (Norwegian) scabies, case report

## Abstract

**Introduction:**

Exfoliative dermatitis, also known as erythroderma, represents an infrequent clinical presentation in emergency departments (ED) and often contributes to considerable diagnostic uncertainty. The differential diagnosis is broad, and the condition may arise from a wide variety of etiologies. We report a case of severe exfoliative dermatitis characterized by gradual progression and resistance to multiple therapeutic interventions over several weeks, ultimately resulting in significant systemic complications and marked hematological abnormalities.

**Case Report:**

A 57-year-old man presented to the ED with severe pain, pruritus, and widespread skin peeling involving more than 90% of his body surface area. The patient developed a rash following a trip to Korea about six months prior to presentation, which progressively worsened despite multiple prior diagnoses and treatments including corticosteroids, immunosuppressive therapy, and biologic agents. On presentation, the patient met systemic inflammatory response syndrome criteria and was found to have profound leukopenia, thrombocytopenia, hypoalbuminemia, and *Staphylococcus aureus* bacteremia. Subsequent evaluation confirmed crusted scabies complicated by secondary bacterial infection.

**Conclusion:**

The case demonstrates the complexity of assessing generalized rash in emergency settings and underscores the importance of a thorough history and physical, as well as maintaining a broad differential diagnosis. It shows both the physical and psychological effects of diagnostic uncertainty on treatment adherence, emphasizes early recognition, and notes systemic complications arising from multifactorial etiologies.

## INTRODUCTION

Erythroderma, also referred to as exfoliative dermatitis, is a severe dermatologic condition characterized by diffuse erythema and scaling involving more than 90% of the body surface area. The condition may result from a variety of underlying etiologies, most commonly inflammatory dermatoses, drug reactions, infections, and malignancies.^1^,^2^ Systemic complications may occur due to disruption of the skin barrier and increased metabolic demand. Reported complications include fluid and electrolyte imbalance, thermoregulatory dysfunction, high-output cardiac failure, and infections resulting in potential cellulitis, bacteremia, and sepsis.^1^,^2^

## CASE REPORT

A 57yearold man with no significant past medical history developed a pruritic rash approximately two weeks after returning from travel to Korea. He was initially diagnosed with hand, foot, and mouth disease and received supportive treatment. Four weeks later, the patient was evaluated by a dermatologist and started on oral prednisone and topical triamcinolone for suspected prurigo nodularis. Although this resulted in partial improvement, the patient subsequently developed multiple pruritic lesions that he frequently excoriated. At that time, laboratory studies were normal except for elevated immunoglobulin E and eosinophils. Due to persistent symptoms, treatment was escalated to dupilumab and upadacitinib. Despite these therapies, the lesions progressed with increasing crusting and pruritus.

Over the following eight weeks, the patient’s pain and pruritus worsened, resulting in two emergency department (ED) visits for pain management. Five days prior to presentation to our ED, the dermatologist noted worsening rash with focal skin thickening in the flexural surfaces of the arms and posterior scalp. Upadacitinib was discontinued, and cyclosporine was initiated. Ivermectin was prescribed for possible crusted scabies; however, the patient did not start the medication. The dermatologist sent him to be hospitalized, and after two days, the patient left against medical advice due to inadequate pain control. He subsequently discontinued all medications and developed severe depression secondary to chronic physical symptoms.

**Image 1:**
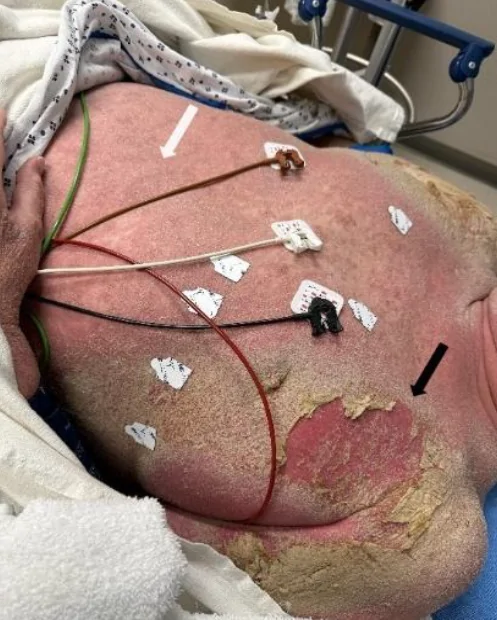
Clinical presentation of exfoliative dermatitis involving the upper body. The patient is shown in a supine position with involvement of the upper extremities and anterior trunk. Diffuse erythema and extensive desquamation are present, with sheets of exfoliating skin in the axillary region exposing the underlying erythematous dermis (black arrow). Multiple scattered blanching and non-blanching petechiae are visible over the chest and extend inferiorly toward the abdomen (white arrow).

**Image 2:**
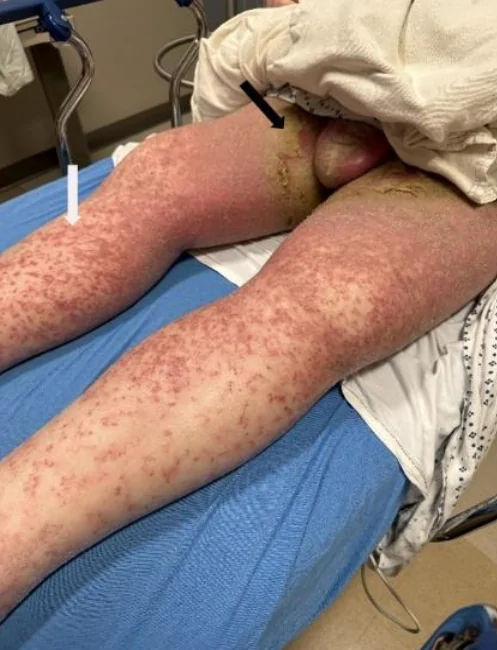
Clinical presentation of exfoliative dermatitis involving the lower body. The patient is shown in a supine position with involvement of the lower extremities and scrotal region. Diffuse erythema and extensive desquamation are present, with sheets of exfoliating skin in the groin region exposing the underlying erythematous dermis (black arrow). Multiple scattered blanching and non-blanching petechiae are visible over both thighs and extend distally toward the lower legs (white arrow).

The patient was brought to our ED by his family with severe pain and diffuse erythema covering more than 90% of the body surface area with extensive peeling and crusting (Images 1 and 2). Vital signs on presentation demonstrated tachycardia and tachypnea with the following values: respiratory rate, 25 breaths per minute; heart rate, 125 beats per minute; blood pressure, 120/81 mm Hg, temperature 99.1 °F, and oxygen saturation, 100% on room air.

Examination revealed a diffuse erythematous rash involving more than 90% of the body surface area including trunk, extremities, scrotum, and buttocks. There was extensive desquamation with sheets of exfoliating dermatitis revealing moist underlying dermis. Thick yellow-crusted plaques were present within intertriginous regions including axillae and groin. Scattered petechiae were present over the distal lower extremities. The conjunctiva and mucous membranes were spared. Nikolsky sign was negative.

The patient met systemic inflammatory response syndrome criteria due to tachycardia and tachypnea. Our sepsis protocol was initiated including intravenous fluid resuscitation and empiric antibiotic therapy with vancomycin and cefepime. Laboratory testing demonstrated profound cytopenias. The white blood cell count was 0.84 × 10^3^ per microliter (μL) (reference range, 4–11 × 10^3^/μL). Hemoglobin was 11.4 grams per deciliter (g/dL) (13.5–17.5 g/dL). Platelet count was 2 × 10^3^ per μL (150–400 × 10^3^/μL). Serum studies demonstrated albumin of 2.0 g/dL (3.5–5.2 g/dL). Initial lactate was 2.7 millimoles per liter (mmol/L) (0.5–2.0 mmol/L) and improved following fluid resuscitation. Urinalysis showed no evidence of infection. Screening for sexually transmitted infections, including *Treponema pallidum (syphilis)*, *Chlamydia trachomatis*, *Neisseria gonorrhoeae*, and *Trichomonas*, was negative. Blood cultures subsequently grew *Staphylococcus aureus*.

Following stabilization and multidisciplinary discussion with dermatology, hematology, and critical care teams, the patient was transferred to a tertiary-care burn center for further management. Ten days later, on follow-up, he was diagnosed with crusted scabies complicated by cellulitis and was treated with ivermectin leading to significant clinical improvement.

*CPC-EM Capsule*

What do we already know about this clinical entity?

*Erythroderma is a rare dermatologic emergency with diverse etiologies and risk of systemic complications requiring rapid recognition.*

What makes this presentation of disease reportable?

*This was an atypical presentation of scabies mimicking inflammatory disease, resulting in progressive erythroderma and systemic decline.*

What is the major learning point?

*Persistent rash with clinical deterioration requires reconsideration of alternative and overlapping etiologies.*

How might this improve emergency medicine practice?

*Early recognition of atypical causes of erythroderma can prevent diagnostic delay and reduce morbidity.*

## DISCUSSION

Initially, the patient was treated for prurigo nodularis, an inflammatory condition characterized by vicious cycles of intense itching and scratching. Although its exact pathogenesis remains unclear, treatment strategies are primarily aimed at interrupting this itch–scratch cycle.^3^–^5^ Treatment options for prurigo nodularis include topical and systemic corticosteroids, with progression to systemic neuromodulating or immunomodulating agents, and phototherapy in selected cases.^3^ Treatment with topical and oral corticosteroids in our patient resulted in partial improvement; however, the lesions did not fully resolve. Following the initiation of immunosuppressive therapy, the patient’s condition progressively worsened, and he ultimately presented to our ED with severe exfoliative dermatitis.

The differential diagnosis of diffuse exfoliative dermatitis is broad, encompassing drug-related, inflammatory, infectious, and malignant etiologies. Moreover, it may be even more complex because of the interaction of multiple etiologies. Crusted (Norwegian) scabies were considered in this patient due to the presence of thick yellow crusting in intertriginous areas, chronicity, immunosuppression, and refractory dermatitis. This condition may lead to secondary bacterial infections, potentially contributing to cytopenias.^6^ Baboon syndrome, also known as symmetrical drug-related intertriginous and flexural exanthema (SDRIFE), remained a plausible diagnosis given the patient’s symmetric flexural involvement, chronic course, absence of mucosal lesions, and possible exposure to systemic immunomodulators. Diagnostic criteria for SDRIFE include systemic drug exposure (first or repeated doses), well-defined erythema on the gluteal/perianal or inguinal areas, involvement of at least one other intertriginous fold, symmetric lesions, and absence of systemic symptoms.^7^

Drug reactions, including Stevens-Johnson syndrome and toxic epidermal necrolysis, classically present with the acute onset of a painful erythematous rash, epidermal necrosis, positive Nikolsky sign, and mucosal involvement, and are often preceded by fever and recent high-risk drug exposure.^8^ In this patient, the absence of mucosal involvement, chronic flexural crusting, and lack of a clearly new offending drug made Stevens–Johnson syndrome and toxic epidermal necrolysis less likely, although the extensive desquamation and cytopenias warranted cautious consideration.

Drug-induced aplastic anemia may manifest with profound leukopenia and thrombocytopenia and occasionally with skin signs such as petechiae and ecchymoses due to thrombocytopenia.^9^ The patient’s cytopenias suggested bone marrow suppression. The presence of petechiae secondary to severe thrombocytopenia indicated that aplastic anemia was a contributing factor to the patient’s skin presentation. Furthermore, the profound leukopenia and resultant immunocompromised state likely predisposed the patient to superimposed bacterial infection and facilitated the severe progression of the parasitic infestation.

Disseminated infection or toxic shock syndrome may show generalized erythema, desquamation, cytopenias, and sepsis. Supporting features include thick crusted plaques, immunosuppression, and *S aureus* growth in blood culture; however, the patient did not have shock physiology or mucosal involvement. Review of the case shows the patient met only two of the five diagnostic criteria for staphylococcal and streptococcal toxic shock syndrome as per the U.S. Centers for Disease Control and Prevention recommendations, although lab criteria was positive.^10^ While the patient did not meet the criteria for toxic shock syndrome, the staphylococcal infection clearly contributed to both the cutaneous and systemic manifestations.

Inflammatory dermatoses, specifically psoriasis and Sézary syndrome, remain clinically significant considerations. Psoriasis may manifest as erythrodermic episodes characterized by widespread scaling and pruritus, whereas Sézary syndrome is typically associated with generalized erythema, lymphadenopathy, and the presence of circulating atypical T cells.^11^–^12^

Ultimately, the patient’s exfoliative dermatitis was attributed to crusted scabies, which became fulminant due to underlying immunosuppression. This predisposed him to a secondary bacterial infection leading to cellulitis, which subsequently progressed to sepsis.

## CONCLUSION

This case illustrates an uncommon presentation in the emergency department and underscores how diverse disease processes can manifest as complex dermatologic emergencies. The combination of immunosuppression, thrombocytopenia, and concurrent bacterial and parasitic infections culminated in exfoliative dermatitis with systemic, potentially life-threatening manifestations. It highlights the diagnostic challenges posed by overlapping multietiologic conditions in dermatology. Maintaining a broad differential diagnosis after a careful history with a thorough review of prior evaluations is essential when managing patients with progressive dermatologic presentations in the emergency setting.
